# To Prevent Ousting

**Published:** 1855-06

**Authors:** 


### TO PREVENT OUSTING.

If you want to go to a church other than your own, and do not want to be marched in and out of the pew two or three times in the course of the service, go early, take a lady with you, ask the sexton for a seat, go in first yourself to the farther end of the pew, and let the lady follow; you will be well paid in the feelings of relief from the annoying apprehensiveness, that every person nearing the pew door is the owner or lady, to whom it is necessary to pay the accustomed deference of getting up and allowing them to pass in.

The history of this valuable discovery of mine may be instructive. In 1843, I happened to be in Philadelphia at the time when persons were returning from the springs and other places of public resort. It was announced in the papers that Dr. Bethune had returned to the city, and would preach next day, (Sabbath); the public were invited to attend. Having a desire to hear the celebrated poet-preacher, I went, taking with me a southern gentleman, an invalid. We went early, to prevent disturbing others, and were shown to a pew in the central block. I was reading a hymn, and on looking up, noticed a man and woman standing at the pew door. I interpreted a nod of the head from the former to mean that he was the owner, and wanted us to come out and let his companion in; accordingly, she passed in and took the seat farthest from the aisle, and he occupied the one next the door; not observing any intimation that we should return, we went to the vestibule and asked the sexton for a seat; he said we could find seats in the gallery, but my friend could not conveniently go up stairs; so we waited in the vestibule until the congregation appeared to be all collected, when we went in again and occupied the bench up against the wall nearest the door, which seemed to be free to all. My friend was by this time so wearied in body, and ruffled in mind, that the sermon did him no good at all. I was sorry for it, because it was the last he ever heard. As for myself, I had become case hardened; intercourse with the world and travel had rhinocerosed my sensibilities, and I employed myself in devising some method of effectually preventing the recurrence of such a *contre temps.* The result was three resolutions:

1. Go to no church but my own.

2. If called occasionally to go to another church, without public invitation, to take the seat without cushions or books nearest the door, usually appropriated to negroes and "poor white folks."

3. If by public invitation, construing it to mean that seats are free to all who come, to take a lady, go early, and pass into the pew before her.

I have found this an unfailing recipe, and it is worth being remembered, if you are modest or ugly and conscious of it, do not like to be seen. If you are handsome and well dressed, take the usual method, and you will have several opportunities of attracting the attention of the whole congregation.

